# A Rare Occurrence of Craniopharyngioma Causing Visual Disturbance in Pregnancy

**DOI:** 10.7759/cureus.18700

**Published:** 2021-10-12

**Authors:** Niki Ho Wai Wye, Ainal Adlin Naffi, Othmaliza Othman, Mae-Lynn Catherine Bastion

**Affiliations:** 1 Ophthalmology, Universiti Kebangsaan Malaysia Medical Centre, Kuala Lumpur, MYS

**Keywords:** suprasellar tumour, visual pathway, optic chiasm, pregnancy, craniopharyngioma

## Abstract

Craniopharyngioma is a rare and benign sellar and suprasellar region tumour. It rarely manifests during pregnancy. We report a 32-year-old primigravida at 19 weeks of gestation with a craniopharyngioma, presenting with visual disturbances during pregnancy. Her vision was 6/9 OD and counting fingers OS with a relative afferent pupillary defect in the left eye. Fundi were normal bilaterally. Visual field testing showed a nasal field defect OD and generalized depression OS. Brain magnetic resonance imaging revealed a suprasellar tumour with chiasmatic compression. Craniotomy and excision of the tumour were done at 20 weeks of gestation. Histopathological examination was consistent with craniopharyngioma. Postoperatively, mother and foetus were stable. Vision improved from counting fingers to 6/6 OS and remained at 6/9 OD. Subsequently, she delivered a healthy baby at term. Such rare and difficult cases warrant close multidisciplinary cooperation pre- and post-operatively to attain optimal outcomes for both mother and baby. By optimizing the patient's medical condition, risks of complications may be reduced. A poor pre-operative vision should also not deter surgical intervention as a proven good visual outcome is achievable.

## Introduction

Craniopharyngioma is a neoplasm composed of cysts lined by stratified squamous epithelium derived from vestigial remnants of the craniopharyngeal anlage [1]. It is a benign tumour of the sellar and suprasellar region. It is rare, with an incidence of 0.13 cases per 100,000 people/year [2]. It comprises 3% of all intracranial tumours and 30% of all new growths in the hypophyseal area [3]. Even more rarely, craniopharyngiomas may manifest during pregnancy. Our case report describes a craniopharyngioma in a pregnant lady who presented with visual disturbance, headache and vomiting with successful visual recovery after tumour excision during the antepartum period, without foetal adverse effects.

## Case presentation

A 32-year-old primigravida at 19 weeks of gestation presented with a left eye painless progressive visual loss for two weeks. She described her left vision as poor, with the nasal field being more pronounced. The right vision was good, but its nasal field appeared dimmer. She experienced headache and vomiting, but without other neurological deficits.

**Figure 1:**
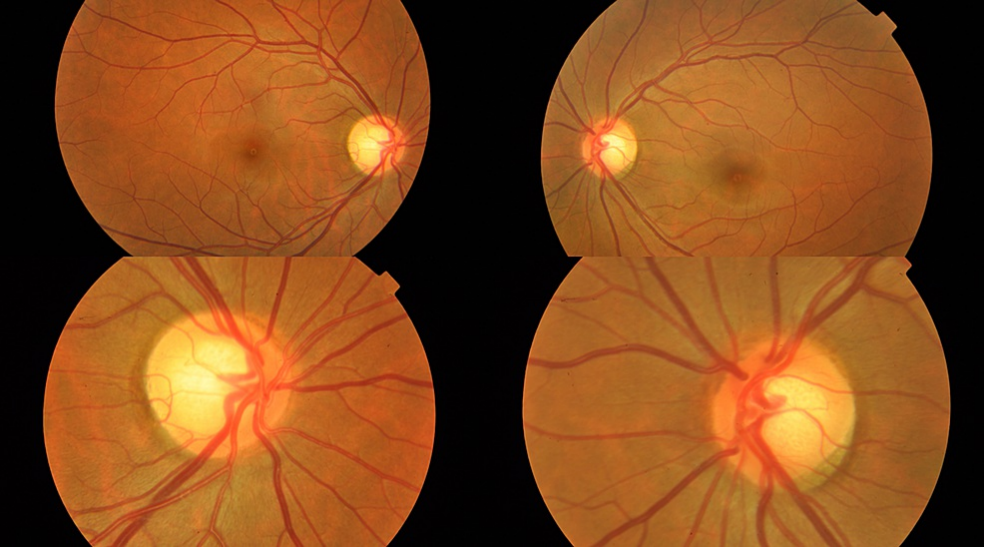
Fundus photos showing normal optic discs and retinae

Her visual acuity was 6/9 OD and counting fingers OS. The relative afferent pupillary defect was present in the left eye. Anterior segment and intraocular pressures were normal. Funduscopy depicted normal optic discs with no papilloedema or optic atrophy. The cup-to-disc ratio was 0.7 bilaterally, with no glaucomatous features. Retinae appeared normal (Figure 1).

**Figure 2:**
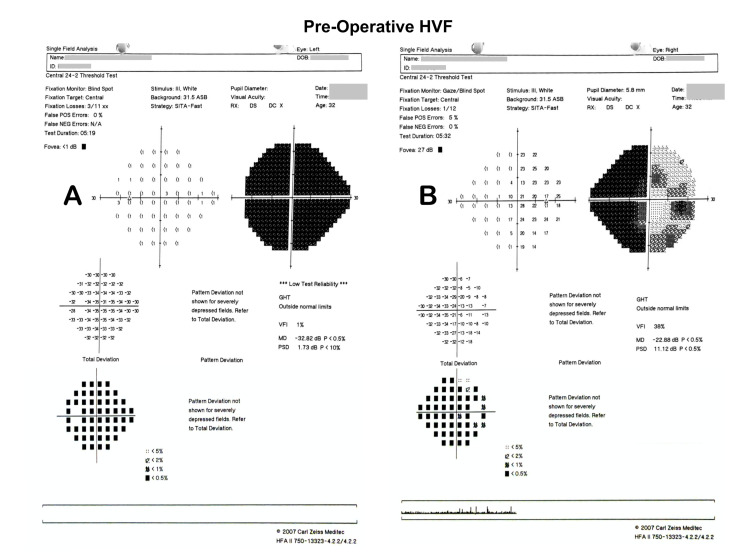
Pre-operative HVF showing generalized depression OS (A) and nasal field defect OD (B) HVF: Humphrey Visual Field

Confrontation test revealed a right eye nasal field defect with poor left vision. Other neurological examinations were unremarkable. A Humphrey Visual Field (HVF) showed a nasal field defect over her right eye and generalized depression over her left eye (Figure 2).

**Figure 3:**
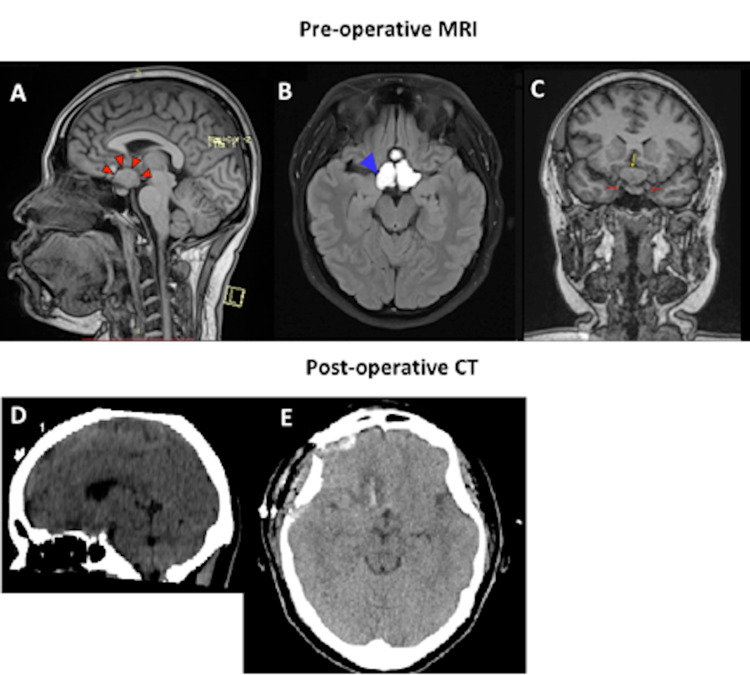
Pre-operative MRI brain showing: (A) Sagittal T1 showing suprasellar lesion (red arrowhead) compressing optic chiasma. The normal pituitary gland is seen, with infundibulum stretched posteriorly by the tumour; (B) Axial FLAIR image showing hyperintense suprasellar mass (blue arrowhead); (C) Coronal T1W showing suprasellar mass (yellow arrow) indenting both ICA (red arrow). Post-operative CT brain showing: (D and E) Plain CT axial and sagittal showing evidence of craniotomy with post-operative changes. Suprasellar mass not visualized. ICA: Internal carotid artery; FLAIR: Fluid-attenuated inversion recovery.

Optical coherence tomography depicted a normal retinal nerve fibre layer thickness. An urgent brain magnetic resonance imaging (MRI) showed a suprasellar tumour with chiasmatic compression, with no radiological signs suggesting raised intracranial pressure (Figure 3).

The diagnosis, treatment options and prognosis of both mother and foetus were discussed between the neurosurgeon, obstetrician, anesthesiologist, endocrinologist, radiologist, and ophthalmologist. The patient and her husband were counseled and consented to surgery.

She underwent craniotomy and excision of the tumour with perioperative steroid administration at 20 weeks of gestation in an attempt to salvage her vision. Intraoperatively, most of the tumour was removed, but some adherent capsule remained. It consisted of a mixture of solid and cystic components. The optic chiasm was decompressed.

**Figure 4:**
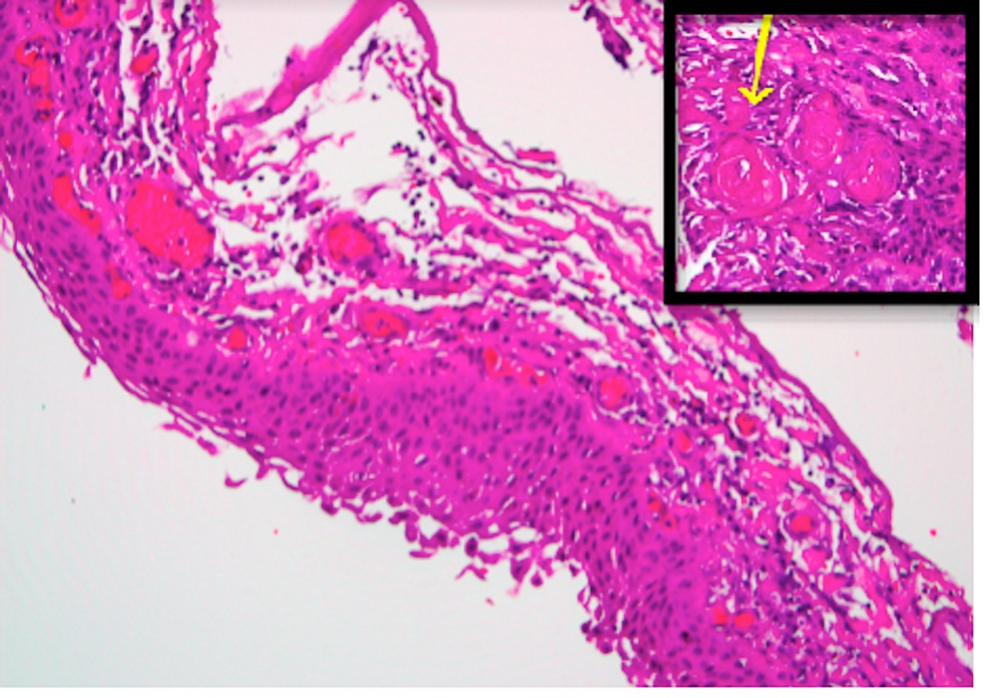
Histopathological examination showing squamous epithelium with areas of wet keratinization (yellow arrow) consistent with craniopharyngioma

Histopathological examination was consistent with craniopharyngioma, showing fibrocollagenous tissue lined by squamous epithelium with keratinization, without palisading columnar cells or stellate reticulum. There was no evidence of malignancy (Figure 4).

**Figure 5:**
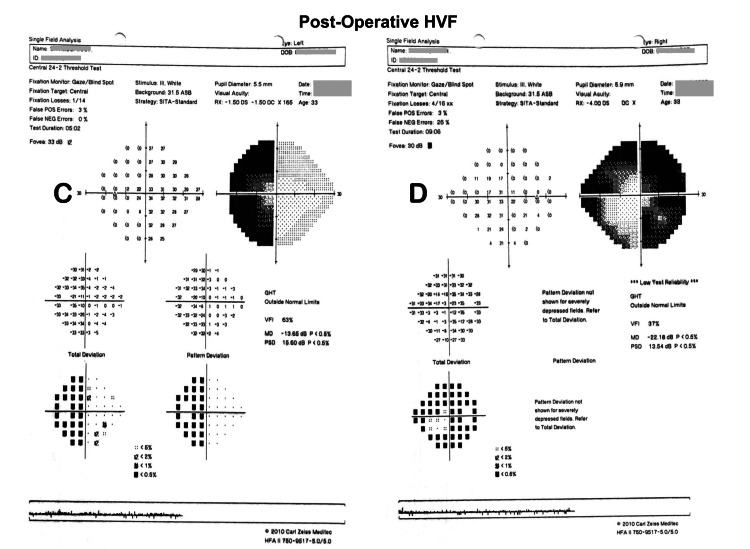
Post-operative HVF showing bitemporal hemianopia, OS (C) and OD (D)

Post-operatively, both mother and foetus were stable. On post-operative day 5, her vision improved from counting fingers to 6/6 OS and remained at 6/9 OD, and nasal field dimness reduced. Post-operative HVF showed bitemporal hemianopia (Figure 5).

She was well and was discharged after five days. Subsequently, her pregnancy progressed normally with a healthy baby boy delivered weighing 3.16kg at term via caesarean section.

## Discussion

In this case, we highlighted several facets of interesting findings. Albeit craniopharyngioma is a rare tumour and even rarer in pregnancy, a handful of cases among expecting mothers were reported, as in our case. Preexisting pituitary adenomas and meningiomas were noted to grow rapidly during pregnancy, but detection of craniopharyngioma during pregnancy is rare, with only a handful of cases reported. Hormonal influences may influence craniopharyngiomas’ growth, but results were inconsistent [4].

With the proximity of these tumours and visual pathway, ocular manifestations are invariably presenting features, which are reduction in visual acuity, visual field defects, optic disc swelling, optic atrophy and squint secondary to cranial nerve sixth palsy. About 49% of craniopharyngioma patients present with ocular manifestations as a presenting symptom [5]. This ocular manifestation is reflected in our patient, who presented with a reduction in visual acuity as well as visual field defects as a presenting symptom of craniopharyngioma.

Visual field disturbances are related to optic chiasm distortion by the tumour. These include mostly bitemporal hemianopia, and in some homonymous hemianopia [5]. Our patient initially presented with a right nasal field defect with generalized depression in the left, which then had a distinct change and became a bitemporal field defect. This phenomenon is called pleomorphism, noted in about 22% of patients [5]. These fluctuations can occur in solid tumours (perhaps due to local oedema) and cystic tumours (from periodic emptying of the cyst into the ventricles) [3].

We postulate that the initial field defect is because of the tumour’s solid cystic constituency, which may cause irregular compression; the left optic nerve being compressed and more compression on the lateral fibres of the right optic chiasm. However, this could not be proven radiologically as the compression appeared generalized.

The mainstay of treatment is complete resection via transsphenoidal or intracranial approach [6]. Precious pregnancies, and our patient being an elderly primigravida with primary subfertility, posed a dilemma during decision-making of surgery during the antepartum period.

There were concerns from different disciplines. Under neurosurgery, early decompression might salvage vision and prevent worsening, but if delayed, risks blindness. The major complications that may arise from this surgery included infection, bleeding, cerebrospinal fluid leak or injury to the pituitary gland, which did not occur in this patient. Glasgow Coma Scale remained full throughout the post-operative period. Monitoring for diabetes insipidus post-operatively was clinically and biochemically normal, with normal urine output, normal serum sodium, urine sodium, serum osmolality and urine osmolality. She was also given some intravenous steroids pre- and post-operatively to reduce cerebral oedema.

Under obstetrics and anesthesiology, there was a risk of miscarriage due to general anaesthesia. This risk of miscarriage was reduced in her case, by giving one dose of intramuscular hydroxyprogesterone caproate 500mg two days prior to surgery. Post-operatively, the obstetric team was involved in fetal monitoring which was done closely in the post-operative period, and there was no miscarriage.

Her hormonal levels were normal, without issues in the endocrinology discipline. Endocrine deficits are frequently caused by disturbances to the hypothalamic-pituitary axes that affect growth hormone secretion, gonadotropins, adenocorticotropic hormone and thyroid stimulating hormone [7]. Incidence range 40-87% of patients with at least one hormonal deficit, and other endocrine symptoms included neurohormonal diabetes insipidus, with a pre-operative incidence of 17-27% [7]. Fortunately, assessment of hormonal levels revealed a normal serum prolactin, thyroid function and cortisol with normal serum sodium, urine sodium, serum osmolality and urine osmolality, indicating no evidence of diabetes insipidus.

With many concerns at hand, it was essential for a multidisciplinary approach, to ultimately be mindful of patient and family decisions.

Majority of craniopharyngiomas in pregnancy had their vision improved after tumour resection, whether resected during pregnancy or postpartum [4]. In one report, the patient presented with a full visual return post-operatively after 48 hours of no light perception [8]. Our case also showed rapid recovery of vision from counting fingers to 6/6, evident in her left eye. Thus, a poor pre-operative vision should not deter surgical intervention in an attempt to restore compromised vision as a very good visual outcome is proven by Maniker and Krieger [8], and our case.

The good visual result is promising in pregnancy with a review of other cases showing vision returning to near normal or normal after tumour resection in at least six out of eight cases (>75%) [4]. In the non-pregnant group, a review of 30 patients showed 42% having a reduction in visual acuity pre-operatively, and 23% with reduced vision post-operatively, with only six out of 25 eyes (24%) showing improvement in visual acuity [9]. The non-pregnant group seems to show less improvement than the pregnant group, but there is little information as to why.

The recurrence rate of craniopharyngioma ranges 40.7-55% [2]. Thus, adequate counselling and follow-up are essential to ensure early detection of recurrence, if present.

## Conclusions

This case showed a very good visual recovery after excision of craniopharyngioma in a pregnant lady and is consistent with other cases, showing a good visual outcome. A multidisciplinary approach is vital in such a rare and challenging case of craniopharyngioma in a pregnant patient for the best possible health outcome for both mother and foetus. By adequately optimizing the condition pre-operatively, we may reduce the risk of certain complications in multiple disciplines such as blindness, high intracranial pressure, risk of miscarriage, endocrine imbalances, etc. Despite a poor pre-operative vision, we should not deter surgical intervention from restoring compromised vision as a proven good visual outcome is achievable. Post-operatively, the patient will also require a long-term follow-up period to ensure early detection of recurrence, if present.
